# The power of social influence: A replication and extension of the Asch experiment

**DOI:** 10.1371/journal.pone.0294325

**Published:** 2023-11-29

**Authors:** Axel Franzen, Sebastian Mader

**Affiliations:** Institute of Sociology, University of Bern, Bern, Switzerland; Lund University: Lunds Universitet, SWEDEN

## Abstract

In this paper, we pursue four goals: First, we replicate the original Asch experiment with five confederates and one naïve subject in each group (N = 210). Second, in a randomized trial we incentivize the decisions in the line experiment and demonstrate that monetary incentives lower the error rate, but that social influence is still at work. Third, we confront subjects with different political statements and show that the power of social influence can be generalized to matters of political opinion. Finally, we investigate whether intelligence, self-esteem, the need for social approval, and the Big Five are related to the susceptibility to provide conforming answers. We find an error rate of 33% for the standard length-of-line experiment which replicates the original findings by Asch (1951, 1955, 1956). Furthermore, in the incentivized condition the error rate decreases to 25%. For political opinions we find a conformity rate of 38%. However, besides openness, none of the investigated personality traits are convincingly related to the susceptibility of group pressure.

## 1. Introduction

A core assumption in sociology is that what humans think and do does not only depend on their own attitudes and disposition, but also to a large extent on what others think and do. The power of social influence on individuals’ behavior was demonstrated already in the 1950s in a series of experiments by Solomon Asch [1–3]. Asch invited individuals into the lab and assigned them the task of judging the length of a line. He also placed 6 confederates into the lab who were assigned to give wrong answers publicly, so that the naïve subject could hear them before he provided his own answer. The results were very surprising: on average 35% of the real subjects followed the opinions of the confederates even if their answer was obviously wrong. The work of Asch has attracted a great amount of attention in the social sciences. Hence, a multitude of replications, extensions, and variations of the original studies have been conducted. However, many of these replications were done with student samples in the US, and fewer studies consist of samples from other countries. Furthermore, many replications were undertaken in the 40 years following the original experiment of Asch, but there are fewer replications thereafter. This raises two important questions: First, are the findings of Asch universal or do they predominantly apply to American students? And second, are the findings still valid today or has the influence of others diminished over time, for instance through increased education and democratization?

Moreover, many experiments in psychology are not incentivized by monetary rewards. This is also true for Asch’s original experiments and for most replications of it. However, in real life outside the lab, decisions are usually associated with consequences, either pleasant in the form of rewards, or unpleasant in the form of some kind of punishment. To make the study of decision-making more realistic, experiments in economics usually use monetary incentives [4]. To provide a conforming but wrong judgment in the original Asch experiment has no consequences, giving rise to the interesting question of whether the finding of Asch still holds when correct answers are rewarded. So far, the effect of incentives in the Asch decision situation has only been investigated rarely [5–7], with inconclusive evidence. Baron et al. [5] report that use of monetary incentives actually increased conformity when the task was difficult. A decreased conformity rate was only found in situations with easy tasks. Bhanot & Williamson [6] conducted two online experiments and found that incentivizing correct answers increases the number of conforming answers. Fujita and Mori [7] compared group reward and individual rewards in the Asch experiment and found that conformity vanished in the individual reward condition. Thus, the existing evidence on the role of incentives is inconclusive, calling for further investigations of the effect of incentives.

Of course, misjudging the length of lines when others do is not important in itself; the Asch experiment created so much attention because it elicits the suspicion that social influence is also present in other and more important social realms, for instance when it comes to political opinions. Early research by Crutchfield [8] suggests that the original findings on line judgment also transfer over to political opinions. We are only aware of one further study by Mallinson and Hatemi [9] that investigates the effect of social influence on opinion formation. However, the authors used a group discussion in the treatment condition, and hence diverged somewhat from the original Asch design. Furthermore, investigations of the effect of social conformity on political opinions are always idiosyncratic making further replications on the transferability from lines to a variety of political opinions important and interesting.

Moreover, behaving in a conforming way and misjudging tasks raises a number of interesting questions. About one third of Asch’s subjects was susceptible to social pressure on average. The rest solved the task correctly irrespective of the confederates’ opinion most of the time. How do those who are not influenced by the group differ from the ones that behave in a conformative manner? Crutchfield [8] investigated a number of personality traits such as competence, self-assertiveness, or leadership ability on the susceptibility to the pressure to conform to the groups’ judgment. However, many of the measurement instruments used by him or by others [10, 11] investigating similar questions are suboptimal, and furthermore produced inconclusive results. Hence, it is worthwhile to further investigate the characteristics of those who conform to social pressure and of those who resist it. We are particularly interested in the Big Five, intelligence, self-esteem, and the need for social approval.

The remainder of the article proceeds in four sections. First, in section two, we present an elaborate literature review of the original Asch experiment, and its various replications. Section three describes how we conducted the replication of the Asch experiment and its variant by using political opinions. Furthermore, we describe how we implemented the incentives, and how we measure the various traits that are presumably related to behavior in the Asch experiment. Section four presents the results and section five concludes and discusses ideas for further research.

## 2. Literature review

In Asch’s [2] original experiment 6 to 8 confederates gathered in an experimental room and were instructed to give false answers in matching a line with the length of three reference lines. An additional uninstructed subject was invited into the experimental room and asked to provide his judgment after the next to last of the confederates. Asch [2] reports a mean error rate of 36.8% of the 123 real subjects in the critical trials in which the group provided the wrong answer. This result was replicated remarkably consistently. Bond and Smith [12] conducted a meta study including 44 strict replications, and report an average error rate of 25%. As with the study by Asch [2], the vast majority of these replications were conducted with male university students in the US. However, more recent studies from Japan [13, 14], and Bosnia and Herzegovina [15] also confirm Asch’s findings. Takano and Sogon [14] found an error rate of 25% in male Japanese university students (n = 40) in groups with 6 to 9 confederates. Mori and Arai [13] used the fMORI technique in which participants wear polarized sunglasses allowing the perception of different lines from the same presentation. The method allows to abandon the use of confederates in the Asch judgment task. They replicated the conformity rate for Japanese female subjects (N = 16) but found no conformity for male subjects (N = 10). Usto et al. [15] found an error rate of 35% in 95 university students of both sexes from Bosnia and Herzegovina with five confederates per group. Other studies also show that subjects are influenced by groups, when the confederates provided their judgments anonymously or with respect to different judgment tasks such as judging the size of circles, completing rows of numbers, or judging the length of acoustic signals [8, 12, 16–21]. More recent studies conducted the Asch experiment also with children [22–25] suggesting that the conformity effect can also be found in preschool children. However, some studies also found age effects, such that younger children conformed to the groups majority judgment, but the effect decreases for adolescents [26, 27]. To summarize, given the results of the literature, we expect to find a substantial conformity rate in the replication of the original Asch line experiment (H_1_).

### 2.1 Monetary incentives

An important extension of the original Asch experiment is the introduction of incentives. In everyday life, decisions are usually associated with consequences. However, in the Asch experiment, as in many other experiments in psychology, decisions or behavior in the lab usually have no consequences, besides of standing out in the laboratory group. This raises questions of the external validity of non-incentivized experiments. Theoretically, it can be expected that correct judgments are less important if they are not incentivized. This could imply that the findings of the Asch experiment are partly methodological artifacts. So far there is only limited and inconclusive empirical evidence with respect to monetary incentives in the Asch experiment. Early studies analysed the role of the perceived societal or scientific importance of the task [20]. Later research incentivized correct answers in various conformity experiments. Andersson et al. [28] report that individual incentives decreased the effect of conformity on the prediction of stock prices. However, Bazazi et al. [29] report the opposite. They found that individualized incentives increase conformity in comparison to collective payoffs in an estimation task. In the study of Baron et al. [5] 90 participants solved two eyewitness identifications tasks (a line-up task and a task of describing male figures) in the presence of two unanimously incorrectly-answering confederates. Additionally, task importance (low versus high) and task difficulty (low versus high) were experimentally manipulated resulting in a 2 x 2 between subject design. Subjects in the high task importance condition received $20 if ranked in the top 12% of participants with regard to correct answers. Subjects in the low task importance condition received no monetary incentive for correct answers. The results of Baron et al. [5] show a conformity rate that closely replicates Asch’s [1–3] finding in the condition without monetary incentives. In the condition including a monetary incentive for correct answers, conformity rates drop by about half to an error rate of 15%. However, this result only emerges in the condition with low task difficulty. For the high task difficulty condition, the opposite effect of monetary incentives was observed. Thus, monetary incentives increased conformity when the task was difficult and decreased conformity in situations with easy tasks. However, one drawback of the study of Baron et al. [5] is a rather low sample size, which might explain the differential effects by experimental condition.

Fujita and Mori [7] analysed the effect of individual vs collective payoff in the Asch experiment. They found that the conformity effect disappears in the individually incentivized condition. However, also this study suffered from low sample sizes since there were only 10 subjects in the individualized minority incentive condition. Furthermore, Fujita and Mori [7] used the fMORI method and report that some subjects might have noticed the trick.

Bhanot and Williamson [6] conducted online experiments (using Amazon Mechanical Turk) in which 391 participants answered 60 multiple-choice trivia-knowledge questions while the most popular answer was displayed at each question. Correct answers were incentivized randomly with $0, $1, $2 or $3 each in a within-subject design, i.e. randomized over trials, not over subjects. Bhanot and Williamson [6] found that monetary incentives increase the proportion of answers that align with the majority. Hence, the studies using incentives yield inconclusive and contradicting results: Particularly, Baron et al. [5] found both an accuracy-increasing and accuracy-decreasing effect of monetary incentives depending on task difficulty. Bhanot and Williamson [6] found an increased conformity rate, and Fujita and Mori [7] found that the conformity bias disappears in the individually incentivized condition. Overall, we follow the economic notion that monetary incentives matter and expect that rewards for nonconformity decrease group pressure (H_2_).

### 2.2 Political opinions

Another critical question is, whether matters of fact can be generalized to matters of attitude and opinion. Crutchfield [8] investigated experimentally the influence of social pressure on political opinions in an Asch-like situation. He found that agreement with the statement “Free speech being a privilege rather than a right, it is proper for a society to suspend free speech whenever it feels itself threatened” was almost 40 percentage points higher in the social pressure condition (58%, n = 50) than in the individual judgment condition (19%, n = 40). Furthermore, he observed a difference of 36%-points if the confederates answer “subversive activities” to the question "Which one of the following do you feel is the most important problem facing our country today? Economic recession, educational facilities, subversive activities, mental health or crime and corruption” as compared to an individual judgment condition (48% vs 12%). However, the results are based on a rather small number of cases and decisions were anonymous, unlike the original design of Asch.

To the best of our knowledge, there is only one further study that experimentally investigates the influence of social pressure on opinions regarding political issues in an Asch-like situation. In the study of Mallinson and Hatemi [9] participants (n = 58) were asked to give their opinion on a specific local political issue before and after a 30–45 minutes face-to-face group discussion (treatment condition). In the control condition subjects received written information that contradicts their initial opinion. They found that in the control condition only 8% changed their initial opinion when provided with further information, while in the treatment condition 38% of subjects changed their opinion. Yet, in this recent study the sample size is also rather small. To sum up, given the results of these two studies, we expect that groups exert influence also on political opinions (H_3_).

### 2.3 Individual differences

Crutchfield [8] was also the first who investigated the relationship between personality traits and the susceptibility to the pressure of conformity. He found that low conformity rates were related to high levels of intellectual competence, ego strength, leadership ability, self-control, superiority feelings, adventurousness, self-assertiveness, self-respect, tolerance of ambiguity, and freedom from compulsion regarding rules. High levels of conformity were observed for subjects with authoritarian, anxious, distrustful, and conventional mindsets. However, no substantial correlation was found for neuroticism. Obviously, Crutchfield’s [8] study is limited by a rather low number of subjects (N = 50). Moreover, the measurement instruments used may be debatable from a contemporary point of view. We are aware of one more recent study with a sufficiently high number of study subjects and more rigid measurement instruments to test the influence of personality traits on conformity in Asch-like situations: Kosloff et al. [19] analysed the association of the Big Five personality traits (agreeableness, conscientiousness, extraversion, neuroticism, and openness) with conformity in public ratings of the humorousness of unfunny cartoons in 102 female college students. Kosloff et al. [19] found that subjects with low neuroticism, high agreeableness, and high conscientiousness scores show high levels of conformity. Extraversion, and openness were not associated with conformity ratings. Beyond that, we are not aware of any more studies that investigate the influence of the Big Five personality traits in the original Asch situation. However, there is evidence that openness is linked to nonconformity. Eck and Gebauer [30] argue that “open people engage in independent thought and, thus, rely little on the conformity heuristic”.

Crutchfield [8] studied the effect of intellectual competence on conformity. He found that higher competence was associated with lower levels of conformity. However, intelligence was measured by the subjective ratings of the experimental staff. Iscoe, Williams, and Harvey [10] exposed high school students (7 to 15 years) to group pressure in an acoustic task (counting metronome ticks), and approximated intelligence by subjects’ school records. They found no correlation of school records with conformity. Uchida et al. [31] studied 12 to 14 year-old high school students and assessed scholastic achievements by their school performance. They report that high achievers conformed less to the majority than low achievers. Hence, results of the effect of intelligence on conformity are inconclusive so far and the existing studies use indirect measures (school grades) but do not measure intelligence directly.

The effect of self-esteem (or self-assertiveness, self-consciousness) on conformity was only investigated in a few studies so far. Kurosawa [11] found no effect on conformity when the decision of the minority subject was preceded by two confederates. In groups of four, confederates’ self-esteem had a negative effect on conformity. Similarly, Tainaka et al. [32] found in a sample of Japanese female students that those with low self-esteem conformed more often in a co-witness task.

In addition, the need for social approval may explain individual differences in conformity behavior. The urge to please others by adhering to social norms is expected to be positively related to conformity, simply because conformity is socially approved in many situations and because of a general tendency among humans toward acquiescence. Once more, Crutchfield [8] provided the first hints of a positive relationship between the need for social approval and conforming behavior in an anonymous Asch situation. However, the measurement instrument he used is debatable. Strickland and Crowne [33] confirmed Crutchfield’s [8] finding in a sample of 64 female students exposed to an Asch-like acoustic judgment task using the Crowne-Marlowe (CM) social desirability scale [34, 35] to gauge the need for social approval. Again, we are not aware of any other more recent study on this aspect. Hence, we investigate the association of the need for social approval using the CM social desirability scale as well as a more recent and supposedly more appropriate instrument to capture the need for social approval [36]. Summarizing, we expect to find a positive association between social approval and conformity (H_4_), and negative associations for intelligence (H_5_) and self-esteem (H_6_). With respect to the Big Five we follow Eck and Gebauer [30] and expect a negative relation between openness and conformity (H_7_).

Finally, Crutchfield [8] also analysed the influence of gender on conformity in a sample of 40 female and 19 male college students (study two). He found that young women show higher conformity rates than young men. Yet, in a third study he found that female college alumnae (N = 50) show lower conformity rates than in study one. Hence, Crutchfield’s [8] findings for the gender effect are inconclusive. However, Bond and Smith [12] report in their meta-analysis higher conformity rates for females. The study by Griskevicius et al. [18] shows that gender-differences in conformity depend on the activation of behavioral motives. Men who were primed to attract a mate revealed more independent judgments than women primed to attract a mate, supposedly because of differing mating preferences in men and women. Therefore, we wonder, whether we can replicate the finding that females are more conformative than males in the Asch experiment.

## 3. Design and method

### 3.1 Procedure and materials

The experiment consisted of three parts. Part 1 was designed to replicate the original Asch experiment. For this purpose, we recruited 210 subjects on the campus of the University of Bern. Informed consent was obtained verbally before participants entered the experimental room. We randomized subjects into two groups. In group one subjects had to judge the length of lines, as in the original Asch experiment. For this purpose, we placed 5 confederates in addition to a naïve subject in a room. The confederates were asked to behave as naïve subjects and entered the room one after the other. The front row of the seats in the experimental room were numbered such that subjects sat next to each other. The naïve subject was always assigned to seat number 5, leaving the last seat to another confederate. First, we presented some instructions to the subjects: “Welcome to our study on decision-making behavior and opinions. This study consists of two parts: In the first part in this room, we ask you to solve a total of 10 short tasks. In the second part in the room next door, we ask you to complete a short questionnaire on the laptop. In total, this study takes about 40 minutes. As compensation for your participation, you will receive 20 Swiss francs in cash after completing the study.” We then presented a reference line to subjects next to three other lines that were numbered 1 through 3 on projected slides. Subjects were asked to judge the length of the reference line by naming the number of the line that corresponds to the reference line in length. We presented 10 such line tasks (see Fig A1 in the S1 Appendix). In the first two trials as well as in trials number 4 and 8, confederates pointed out the correct lines. Four trials were easy tasks, since the difference between the reference line and two of the other lines was large. The other six trials were more difficult, since the differences were small. Subjects were asked to call out the number of the correct line always starting with subject 1 through 6.

After the line task in part 2 of the experiment subjects were confronted with 5 general questions on different political issues. The statements were selected because we believe they describe fundamental attitudes towards different political or social groups in a democracy. The five statements read (1) “Do you think that the Swiss Federal Government should be given more power?”, (2) “Do you think trade unions should be given more power in Switzerland?”, (3) “Do you think that the employers’ association in Switzerland should be given more power?”, (4) “Do you think that citizens should be given more liberties in Switzerland?”, and (5) “Do you think that companies in Switzerland should be given more freedom?”. Subjects were asked to answer all 5 questions with either yes or no. The confederates in this group were instructed to answer “yes” to the first question and “no” to the rest. We chose this sequence of “yes” and “no” to prevent that subjects discover the existence of confederates. Finally, part 3 of the experiment consisted of an online questionnaire which subjects were asked to complete. To conceal that some participants were confederates all 6 participants were accompanied to separate rooms where the online-questionnaire was installed on a laptop. The questionnaire was designed to measure a number of different personality traits. Particularly, we measured the Big Five using a 10-item scale (two items for each of the 5 traits) as suggested by Rammstedt et al. [37] (see Table A1 in the S1 Appendix for item wording); a 10-item scale measuring self-esteem as suggested by Rosenberg [38] (see Table A2 in the S1 Appendix); a short version of the Hagen Matrices Test [39] to measure intelligence and the 10-item version of the Martin Larson Approval Motivation Scale (MLAM) [36].

In group 2 the experimental design and procedure was the same as in group 1 besides the fact that correct answers in the length of lines judgment task were incentivized. In addition to the 20 Swiss francs show-up fee, subjects received one Swiss franc for every correct answer in the line judgment task, and hence, could earn up to 30 Swiss francs in total. Since there are no correct answers to political opinions these were not incentivized. However, we randomized the confederates’ answers to political opinion questions independently of whether a subject was in the incentivized or non-incentivized group. In one version confederates answered “yes” to the first question and “no” to the four other questions. In the other version the sequence of the confederates’ response was “no” to the first question and “yes” in response to the other four. The experiment was conducted by three different research teams consisting of 7 student assistants each. In every group 5 students acted as confederates and 2 as research assistants, recruiting subjects, welcoming and instructing them in the laboratory room, and reading out loud the projected instructions.

### 3.2 Sample

A power analysis suggested that we need about 100 subjects per experimental condition to find statistically significant (α = 0.05) differences of 5 percentage points for a power of 0.8. Hence, we stopped recruiting subjects after reaching 210 participants. The experiment was conducted between March 16, 2021 and April 30, 2021. The authors had no access to any information that links individual identifiers to the data. Subjects were debriefed after the end of the study by email.

## 4. Results

Overall, 210 subjects participated in the experiment (female = 61%, mean age = 22.6). 102 subjects were randomly assigned to the non-incentivized group and 108 into the group with incentives. Moreover, 113 subjects were assigned to the sequences of “yes” and four “no” of the political opinion task and 97 to the reversed sequence, suggesting that the randomization procedure worked well. The questionnaire also contained an attention check. The question reads “In the following we show you five answer categories. Please do not tick any of the answers”. Four subjects failed to comply and ticked an answer, suggesting that they did not pay proper attention to the question wording. These subjects were excluded from the analysis. Furthermore, we asked subjects at the end of the questionnaire what they think the experiment was about. Three subjects recognized that the experiment was the line task experiment of Asch or expressed the suspicion that some of the other group members were confederates. We also excluded these three subjects from the analysis. Moreover, one subject answered the question about their gender with “other” and was also excluded from the analysis. Hence, these exclusions result in 202 valid cases. However, the results presented do not depend on these eight excluded observations.

**Fig 1:**
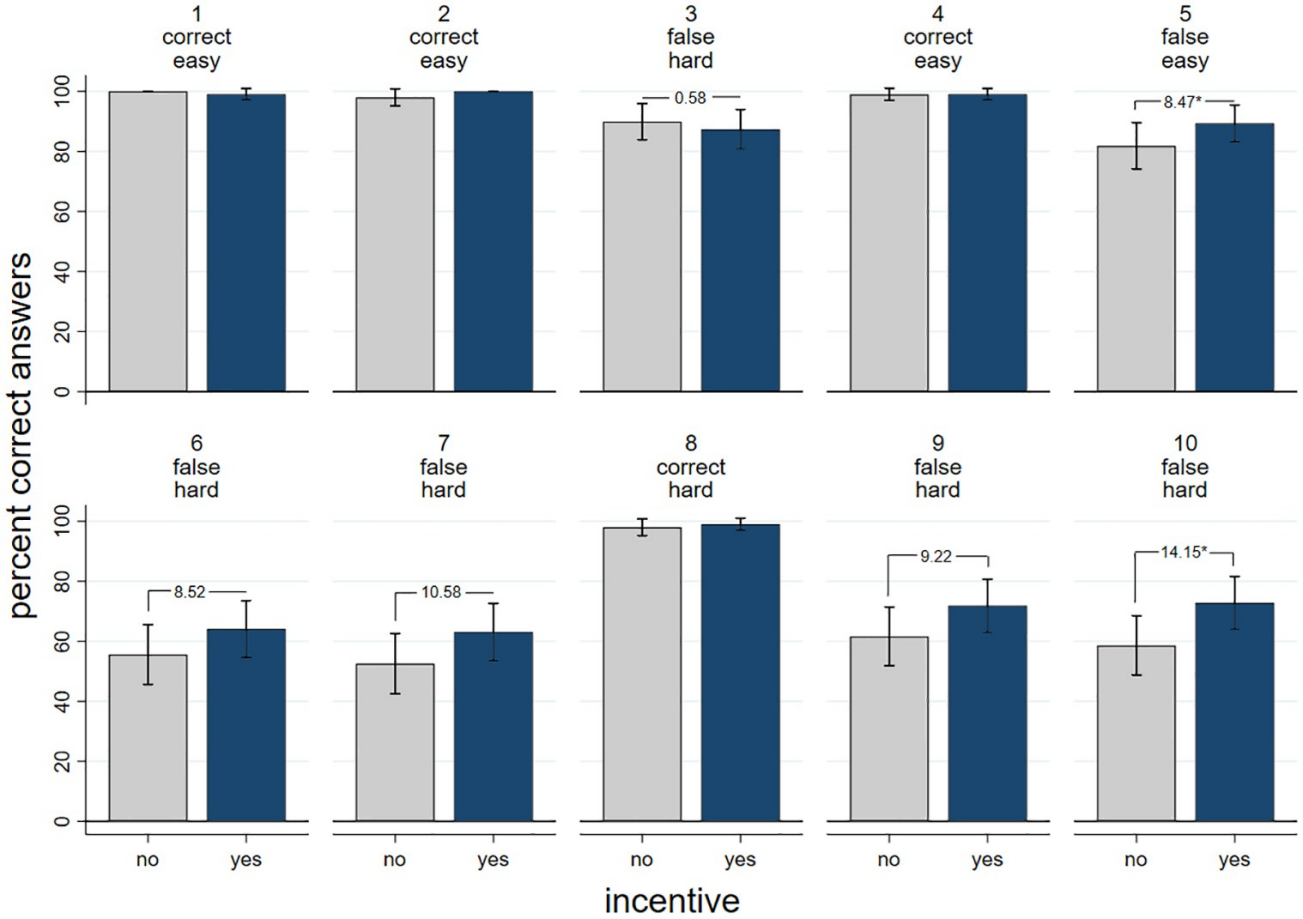
Asch line task: Correct answers by condition and trial. Note: Percent of correct answers by experimental group and trial including 95% confidence intervals. The numbers on top of the bars denote the trial numbers. “correct” stands for uncritical trials, “false” for critical trials. “easy” denotes easy trials with big differences between the lines, and “hard” denotes more difficult trials with smaller differences between the lines. The numbers between the bars denote the difference in proportions between the groups in percentage points. One-sided T-tests: * = *p* < 0.05. N without incentive (no) = 99, n with incentive (yes) = 103.

Fig 1 presents the results of the ten line length tasks for the non-incentivized (grey bars) and for the incentivized conditions (blue bars). As can be clearly seen, almost none of the naïve subjects gave an incorrect answer when the group provided the correct answer which was the case in decision situations 1, 2, 4 and 8. However, when the group provides the false answer a substantial number of naïve subjects provided this incorrect answer as well (decision situations 3, 5, 6, 7, 9, and 10). The proportion of incorrect answers in the non-incentivized condition is relatively small in decision 3 (10%), but relatively high in decisions number 6 and 7 (44% and 47%). The average of incorrect answers is 33% in the non-incentivized group, which is a perfect replication of Asch’s (1955) original 36.8% result (two sample two-sided T-test, t(16) = 0.59, p = 0.57).

When correct answers are incentivized, the proportion of incorrect answers decreases by on average 8%-points. The difference between the groups is statistically significant in 2 out of 6 critical trials (p < 0.05 for one-sided T-tests). The difference also becomes evident when we consider the number of incorrect answers in the 6 critical trials. When decisions were not incentivized subjects gave on average 1.97 incorrect answers. In the incentivized condition the average number dropped to 1.47, leading to a statistically significant difference of 0.5 incorrect answers (t(208) = 2.24, p = 0.03 for two-sided T-test).

**Fig 2:**
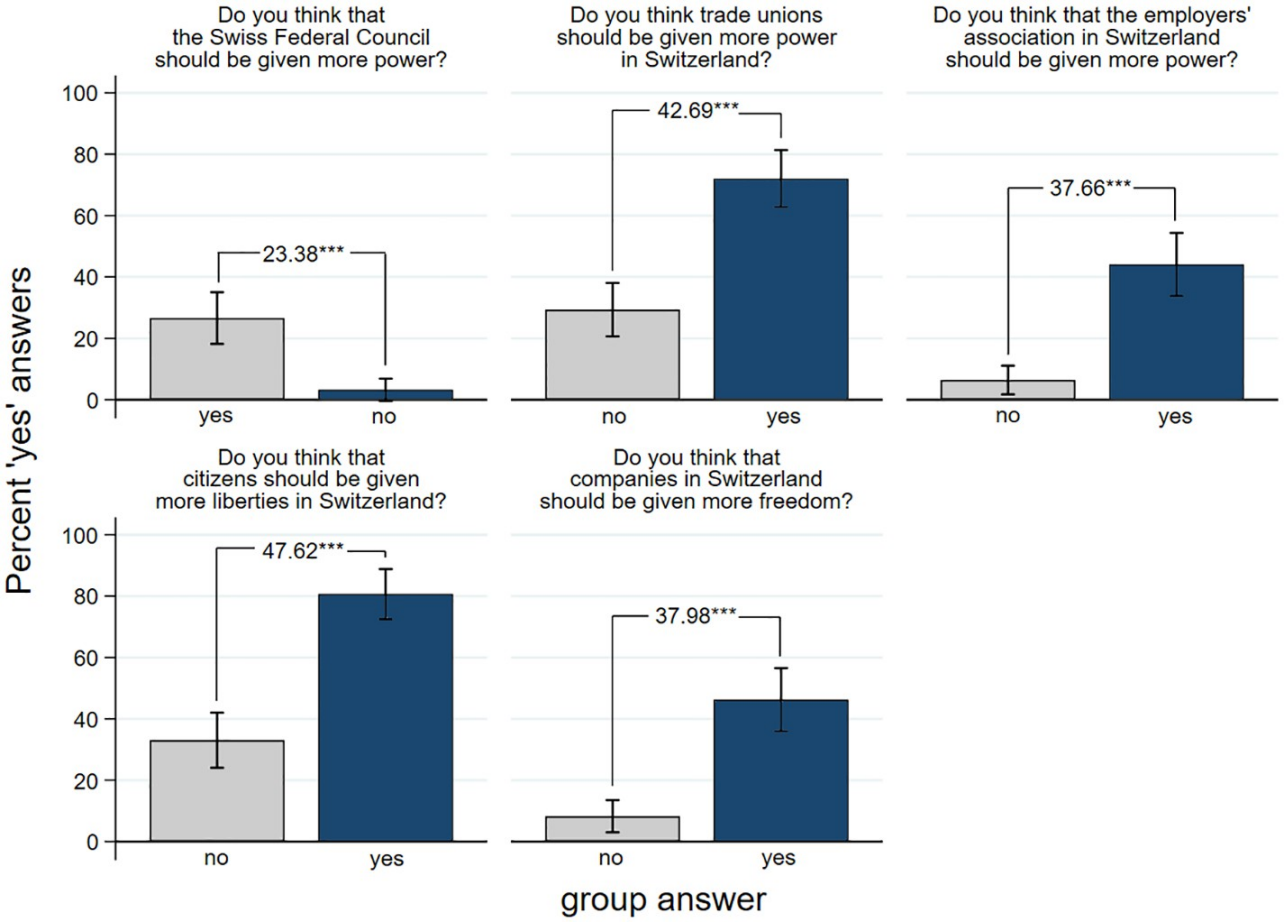
Political opinions and social influence. Note: Percent of ‘yes’ answers to five general questions on political opinions in which all confederates answered uniformly ‘yes’ or ‘no’, by experimental group including 95% confidence intervals. The numbers on top of the bars stand for the difference in proportions between the respective groups in percentage points. Two-sided T-tests: *** = *p* < 0.001. n (sequence yes, no, no, no, no) = 109, n (sequence no, yes, yes, yes, yes) = 93.

Next, Fig 2 presents the results concerning the five political questions. When the group said “yes” to the question of whether the Swiss Federal Council (the government in Switzerland) should have more power, 27% of the naïve subjects did so as well. When the group said “no” only 3% of the subjects said “yes” resulting in a difference of 23.4%-points. When the group said that trade unions should have more power 72% of the subjects answered “yes” as compared to only 29% when the group said “no” resulting in a difference of 43%-points. Similarly, the question of whether the employers’ association should have more power is agreed to by 44% and 6% respectively, depending on the group agreeing or disagreeing. Moreover, 81% of the subjects agreed that citizens in Switzerland should be given more liberties when the group does so, and 33% agreed to this question when the group says “no”. Finally, 46% said that companies should be given more freedom when the group agreed but only 8% did so when the group denied this question. The average difference in the proportion of yes-answers is 38%-points and all 5 differences are statistically highly significant. This result corresponds astonishingly close to the result in the length of line experiment and suggests that the influence of group pressure can be generalized to the utterance of political opinions.

One interesting question is whether the susceptibility to group pressure is linked to certain personality traits. To investigate this question, we count the number of wrong answers in the six critical trials of the length of line task. This variable is our dependent variable and runs from 0 when a subject always gave correct answers to 6 for subjects who gave only wrong answers. First, we wondered whether conformity is linked to the Big Five personality traits. We measured the Big Five using a short 10-item version as suggested by Rammstedt et al. [37] which measures each trait (openness, extraversion, agreeableness, conscientiousness, and neuroticism) with two questions (see Table A1 in the S1 Appendix).

Second, we incorporate a 10-item measure of self-esteem, as suggested by Rosenberg [38], into the analysis (see Table A2 in the S1 Appendix). Each item of the scale has four answer categories ranging from 1 = “disagree strongly”, 2 = “disagree”, 3 = “agree” to 4 = “agree strongly”. Subjects that score high on self-esteem are expected to have stronger confidence in their own perception and should be less influenced by the group’s opinion. Third, we measured individuals’ intelligence using a short version of the Hagen Matrices Test (HMT) [39]. The HMT consists of six 9-field matrices that show graphical symbols that follow a logical order. The last field is missing and the task of the subjects is to pick the correct symbol, out of eight, that fits and completes the pattern of the matrix. Hence, the HMT ranges from 0 if no answer is correct to 6 for subjects who provided six correct answers. The hypothesis is that subjects who score high on the HMT are less susceptible to the pressure of the group and also provide more correct answers in the line task.

**Fig 3:**
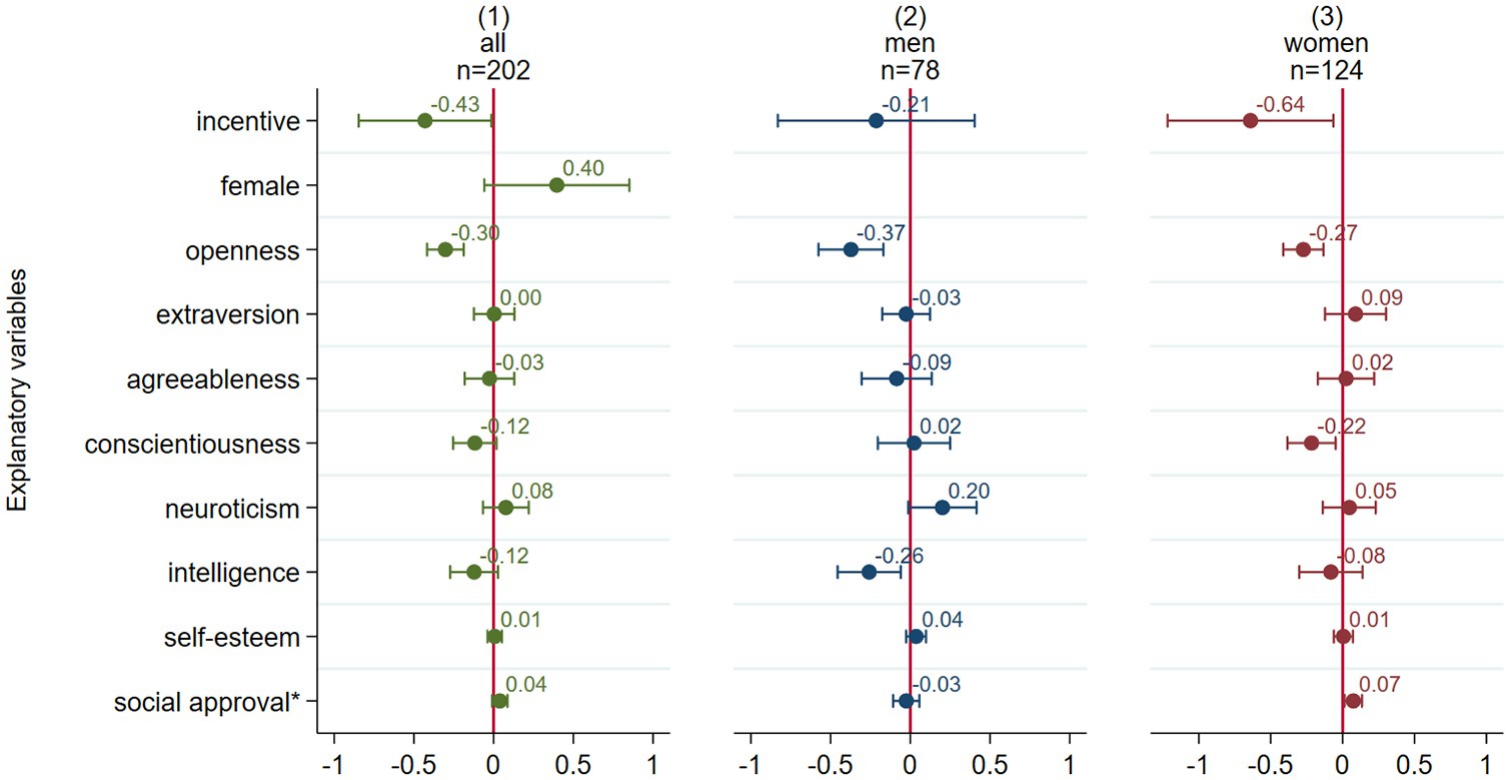
Coefficient plot of individual characteristics on the number of conforming answers (OLS regression). Note: N = 202. Unstandardized coefficients of multiple linear OLS regressions including robust 95% confidence intervals. Poisson and negative binomial models do not alter the results in any substantial way. Variables marked with an ‘*’ indicate statistically significant differences in the coefficients between models (2) and (3).

Finally, conformity might be linked to the need for social approval. We measured the need for social approval with a 10-item version of the Martin Larson Approval Motivation Scale (MLAM) [36] (see Table A3 in the S1 Appendix). Individuals that score highly on the MLAM display high need for social approval by others. Hence, we expect that subjects with higher values on the MLAM should also conform more often to the opinions of others in order to receive social approval. A summary of the descriptive information of the considered variables is depicted in Table A4 in the S1 Appendix. To investigate whether any of the measured personality traits are linked to the answering behavior in the line task we conducted multiple OLS regression analysis. The results of this analysis are depicted in the coefficient plots in Fig 3 (see also Table A5 in the S1 Appendix).

First, model 1 presents the effects on the number of conforming answers for the whole sample. In the incentivized condition subjects gave on average 0.43 fewer conforming answers as compared to the unincentivized condition. This effect mirrors the bivariate result already presented in Fig 1 and is statistically significant for the 5% level. In tendency, females show more conforming answers, but this effect is statistically only significant for the 10% level. Besides “openness” none of the personality traits contained in the Big Five show any statistically significant effects. This is also true for the other effects of intelligence, self-esteem, and the measure for social approval seeking. Models 2 and 3 show the results for men and women separately. The separate results suggest that women react somewhat more strongly to incentives than do men. However, a test for differences in coefficients suggests that the effects do not differ (χ^2^(1) = 1.11, p = 0.29). Intelligence seems to have greater importance for men, leading to 0.27 fewer conforming answers for every correct answer of the HMT. However, this effect does not differ statistically from the effect for females (χ^2^(1) = 1.35, p = 0.25). No difference in effects can be observed for self-esteem. However, in the female sample the need for social approval is positively linked to the number of conforming answers, which is not the case in the male sample (χ^2^(1) = 4.23, p = 0.04); but the effect of social approval in the female sample is relatively small.

We conducted a number of robustness checks with the presented analyses. Since our dependent variable is a count variable (number of conforming answers) the models can also be estimated using Poisson regressions or negative-binomial models. However, none of our presented results change in any substantial way using these alternatives. Furthermore, we excluded 24 more subjects who when asked at the end of the experiment about the goal of the study said that the experiment was about group pressure or conformity, although they did not explicitly mention Asch or the suspicion that other participants were confederates. But these additional exclusions also did not change the results substantially (see Table A6 in the S1 Appendix). Finally, we also incorporated the 10-item version of the Marlowe-Crowne Scale [34, 35] suggested by Clancy [40, 41]. However, inclusion of the scale did not show any statistically significant effects or did change any of the other estimates.

## 5. Conclusion and discussion

In this study we first replicated the original experiment of Asch [1–3] with 5 confederates and ten line tasks. We find an average error rate of 33% which replicates the original findings of Asch very closely and which is in line with other replications that were conducted predominately with American students [12]. Together with recent studies from Japan [14], and Bosnia and Herzegowina [15], our study provides further evidence that the influence of groups on individuals’ judgments is a universal phenomenon, and is still valid today. Furthermore, we incentivized the decisions and find a drop of the error rate by 8%-points to 25%. Hence, monetary incentives do not eliminate the effect of group pressure. This finding sheds doubt on former results which predominately show the opposite effect, namely that incentives increase compliance [5, 6].

Moreover, our study suggests that group pressure is not only influential in the simple line task but also when it comes to political opinions. We randomized the groups’ response to five different political statements and find an average conformity rate of 38%. Hence, these results suggest that the original finding of Asch can also be generalized to matters of opinion. This result is in line with former evidence by Crutchfield [8], and Mallinson and Hatemi [9]. However, both of these studies had only small sample sizes of 50 and 58 subjects respectively, which called for further replication studies. Finally, we measured the Big Five, intelligence, self-esteem and social approval. With the exception of openness, our study finds no support that these personality traits are statistically significantly related to the susceptibility of group pressure.

Of course, our study has some limitations, which suggest a number of further research questions. First, we used a relatively large sample of 202 subjects providing more statistical power than former replications and extensions of the Asch experiment; however, our subjects were also students, and hence, it would be important to have further replications with non-student samples. This would allow further investigations of the susceptibility to group pressure with respect to age, different occupational groups, different social backgrounds, and different levels of social experience.

Second, the subjects we investigate are strangers. That means the single naïve subjects did not know the confederates. An interesting question for further research would be, whether group pressure is stronger among non-strangers or whether dissent becomes more acceptable among a group of friends.

Third, we demonstrate that monetary incentives reduce the error rate. However, our incentives were one Swiss franc for every correct answer, and hence small. Thus, the interesting question remains whether larger incentives reduce the error rate further, or can even lead to the elimination of it.

Fourth, the political statements we choose are relatively moderate and general. This leaves the question open as to whether subjects would also conform to more extreme or socially less acceptable statements. Furthermore, our subjects might have rarely thought about the statements we provided, leaving the question of what would happen with respect to statements about which subjects had stronger opinions or which are more related to their identity.

With the exception of openness all personality traits considered (e.g. intelligence, self-esteem, need for social approval) are not related to conformity. This raises a number of very interesting research questions. One possibility is that the traits were not measured good enough, and that measurement errors impede the identification of these individual differences. This concern applies particularly to the measurement of the Big Five where we relied on the short 10-item version suggested by Rammstedt et al. [37]. Hence, the puzzling result that openness leads to less conformity must be replicated before it can count as a reliable finding. However, the finding is in line with the assumption of Eck and Gebauer [30]. Another possibility is that other personality traits are more important when it comes to conformity behavior. Hence, there is much room for further interesting research concerning conformity behavior in situations of group pressure.

## Supporting information

S1 Appendix (DOCX)
