# Supplementary material for: The power of social influence: A replication and extension of the Asch experiment
Source: PLoS One. 2023 Nov 29;18(11):e0294325. doi: 10.1371/journal.pone.0294325 (PMC10686423; doi:10.1371/journal.pone.0294325)
Supplement: S1 Appendix — (DOCX) [file pone.0294325.s001.docx]

Appendix

(please insert Figure A1 here)

Figure A1: The ten line judgment tasks.





Table A1: Big Five Inventory 10 (BFI-10) [37]

| Openness | |  | *r*=0.33 |
| --- | --- | --- | --- |
| (1) | I see myself as someone who has few artistic interests. | - | 0.82 |
| (2) | I see myself as someone who has an active imagination. | + | 0.82 |
| Extraversion | |  | *r*=0.62 |
| (1) | I see myself as someone who is reserved. | - | 0.90 |
| (2) | I see myself as someone who is outgoing, sociable. | + | 0.90 |
| Agreeableness | |  | r=0.08 |
| (1) | I see myself as someone who is generally trusting. | + | 0.73 |
| (2) | I see myself as someone who tends to find fault with others. | - | 0.73 |
| Conscientiousness | |  | *r*=0.39 |
| (1) | I see myself as someone who tends to be lazy. | - | 0.83 |
| (2) | I see myself as someone who does a thorough job. | + | 0.83 |
| Neuroticism | |  | *r*=0.41 |
| (1) | I see myself as someone who is relaxed, handles stress well. | - | 0.84 |
| (2) | I see myself as someone who gets nervous easily. | + | 0.84 |
| N | |  | 202 |

Note: Numbers indicate factor loadings after varimax-rotated principal component factor analysis per Big Five dimension. *r* = Pearson’s correlation coefficient of the 2 respective items per Big Five dimension. Each item contains four answer categories ranging from 1 = “disagree strongly” to 5 = “agree strongly” for the items indicated by ‘+’. Items with ‘-‘ are reversely scored.

Table A2: Self-esteem [38]

|  |  |  | Self-esteem |
| --- | --- | --- | --- |
| I | (1) | On the whole, I am satisfied with myself. | 0.62 |
|  | (2) | At times I think I am no good at all. | 0.77 |
|  | (3) | I feel I do not have much to be proud of. | 0.53 |
|  | (4) | I certainly feel useless at times. | 0.76 |
|  | (5) | I wish I could have more respect for myself. | 0.74 |
|  | (6) | All in all, I am inclined to feel that I am a failure. | 0.78 |
|  | (7) | I take a positive attitude toward myself. | 0.71 |
| II | (8) | I feel that I have a number of good qualities. | 0.80 |
|  | (9) | I am able to do things as well as most other people. | 0.73 |
|  | (10) | I feel that I'm a person of worth, at least on an equal plane with others. | 0.72 |
|  | N | | 202 |
|  | Cronbach’s α | | 0.86 |
|  | Self-esteem: mean  sd  min, max | | 37.47  4.96  21, 45 |

Note: Numbers indicate factor loadings after varimax-rotated principal component factor analysis. Each item contains four answer categories ranging from 1 = “disagree strongly”, 2 = “disagree”, 3 = “agree” to 4 = “agree strongly” for items 1, and 7 to 10. Items 2 to 6 are reversely scored.

Table A3: The short Martin Larson Approval Motivation Scale (MLAM) [36]

|  |  |  | MLAM |
| --- | --- | --- | --- |
| I | (1) | I would rather be myself than be well thought of. | 0.50 |
|  | (2) | I change my opinion (or the way that I do things) in order to please someone else. | 0.72 |
|  | (3) | In order to get along and be liked, I tend to be what people expect me to be. | 0.82 |
|  | (4) | If there is any criticism or anyone says anything about me, I can take it. | 0.46 |
|  | (5) | It is not important to me that I behave “properly” in social situations. | 0.51 |
| II | (6) | I find it difficult to talk about my ideas if they are contrary to group opinion. | 0.61 |
|  | (7) | I am careful at parties and social gatherings for fear that I will do or say things that others won’t like. | 0.34 |
|  | (8) | I usually do not change my position when people disagree with me. | 0.82 |
| III | (9) | I am willing to argue only if I know that my friends will back me up. | 0.64 |
|  | (10) | I seldom feel the need to make excuses or apologize for my behaviour. | 0.66 |
|  | N | | 202 |
|  | Cronbach’s α | | 0.67 |
|  | Social Approval: mean  sd  min, max | | 24.14  5.01  14, 40 |

Note: Numbers indicate factor loadings after varimax-rotated principal component factor analysis. Each item contains five answer categories ranging from 1 = “disagree strongly” to 5 = “agree strongly” for items 2, 3, 5, 6 and 8, and 1 = “agree strongly” to 5 = “disagree strongly” for items 1, 4, 7, 9 and 10.

Table A4: Description of variables

| Variable | Description | mean | sd | min., max. | n |
| --- | --- | --- | --- | --- | --- |
| Number of conform answers |  | 1.71 | 1.62 | 0, 6 | 202 |
| Gender: female | Dummy. | 0.61 |  | 0, 1 | 202 |
| Big Five Personality Traits | Big Five Inventory 10 (BFI-10; [37], 10 items, 2 per trait) |  |  |  |  |
| Openness |  | 7.32 | 1.80 | 2, 10 | 202 |
| Extraversion |  | 7.18 | 1.69 | 3, 10 | 202 |
| Agreeableness |  | 6.87 | 1.49 | 2, 10 | 202 |
| Conscientiousness |  | 7.03 | 1.66 | 2, 10 | 202 |
| Neuroticism |  | 5.96 | 1.71 | 2, 10 | 202 |
| Intelligence | Short version of the Hagen Matrices Test (HMT-S; [39], 6 items) | 4.36 | 1.44 | 0, 6 | 202 |
| Self-esteem | 10 item scale [38]; Cronbach’s α = 0.86. | 37.47 | 4.96 | 21, 45 | 202 |
| Social Approval | 10 items of the Martin Larson Approval Motivation Scale (MLAM [36]  Cronbach’s α = 0.67. | 24.14 | 5.01 | 14, 40 | 202 |

Table A5: Individual differences in the number of conform answers

| Model | (1) | (2) | (3) |
| --- | --- | --- | --- |
|  | all | men | women |
| Incentivization | -0.43* | -0.21 | -0.64* |
|  | (0.21) | (0.31) | (0.29) |
| Gender: female | 0.40^+^ |  |  |
|  | (0.23) |  |  |
| Big Five: |  |  |  |
| Openness | -0.30*** | -0.37*** | -0.27*** |
|  | (0.06) | (0.10) | (0.07) |
| Extraversion | 0.00 | -0.03 | 0.09 |
|  | (0.06) | (0.08) | (0.11) |
| Agreeableness | -0.03 | -0.09 | 0.02 |
|  | (0.08) | (0.11) | (0.10) |
| Conscientiousness | -0.12^+^ | 0.02 | -0.22* |
|  | (0.07) | (0.11) | (0.08) |
| Neuroticism | 0.08 | 0.20^+^ | 0.05 |
|  | (0.07) | (0.11) | (0.09) |
| Intelligence | -0.12 | -0.26* | -0.08 |
|  | (0.08) | (0.10) | (0.11) |
| Self-esteem | 0.01 | 0.04 | 0.01 |
|  | (0.02) | (0.03) | (0.03) |
| Social approval | 0.04 | -0.03 | 0.07* |
|  | (0.02) | (0.04) | (0.03) |
| Constant | 3.78** | 4.16 | 2.99 |
|  | (1.39) | (2.54) | (1.90) |
| n | 202 | 78 | 124 |
| adj. R^2^ | 0.17 | 0.26 | 0.15 |

Note: Unstandardized coefficients of multiple linear OLS regressions including robust standard errors in parentheses. + p<0.10, * p<0.05, ** p<0.01, *** p<0.001. Poisson and negative binomial models do not alter the results in any substantial way.

Table A6: Individual differences in the number of conforming answers (excluding critical cases)

| Model | (1) | (2) | (3) |
| --- | --- | --- | --- |
|  | all | men | women |
| Incentivization | -0.56* | -0.44 | -0.67* |
|  | (0.22) | (0.29) | (0.31) |
| Gender: female | 0.64** |  |  |
|  | (0.23) |  |  |
| Big Five: |  |  |  |
| Openness | -0.31*** | -0.38*** | -0.28*** |
|  | (0.06) | (0.09) | (0.08) |
| Extraversion | 0.00 | -0.01 | 0.07 |
|  | (0.07) | (0.06) | (0.12) |
| Agreeableness | 0.02 | -0.01 | 0.04 |
|  | (0.08) | (0.10) | (0.11) |
| Conscientiousness | -0.13+ | -0.07 | -0.18+ |
|  | (0.07) | (0.11) | (0.09) |
| Neuroticism | 0.02 | 0.11 | 0.04 |
|  | (0.07) | (0.09) | (0.11) |
| Intelligence | -0.14+ | -0.28** | -0.08 |
|  | (0.08) | (0.10) | (0.12) |
| Self-esteem | 0.01 | 0.02 | 0.01 |
|  | (0.02) | (0.03) | (0.04) |
| Social approval | 0.04 | -0.04 | 0.08* |
|  | (0.03) | (0.04) | (0.03) |
| Constant | 3.89** | 5.68** | 2.51 |
|  | (1.43) | (2.02) | (2.24) |
| n | 178 | 71 | 107 |
| adj. R^2^ | 0.19 | 0.29 | 0.13 |

Note: Unstandardized coefficients of multiple linear OLS regressions including robust standard errors in parentheses. + p<0.10, * p<0.05, ** p<0.01, *** p<0.001. Poisson and negative binomial models do not alter the results in any substantial way.
